# Roles of Chronic Low-Grade Inflammation in the Development of Ectopic Fat Deposition

**DOI:** 10.1155/2014/418185

**Published:** 2014-07-21

**Authors:** Lulu Liu, Mei Mei, Shumin Yang, Qifu Li

**Affiliations:** Department of Endocrinology, The First Affiliated Hospital of Chongqing Medical University, No. 1 Youyi Street, Chongqing 400016, China

## Abstract

Pattern of fat distribution is a major determinant for metabolic homeostasis. As a depot of energy, the storage of triglycerides in adipose tissue contributes to the normal fat distribution. Decreased capacity of fat storage in adipose tissue may result in ectopic fat deposition in nonadipose tissues such as liver, pancreas, and kidney. As a critical biomarker of metabolic complications, chronic low-grade inflammation may have the ability to affect the process of lipid accumulation and further lead to the disorder of fat distribution. In this review, we have collected the evidence linking inflammation with ectopic fat deposition to get a better understanding of the underlying mechanism, which may provide us with novel therapeutic strategies for metabolic disorders.

## 1. Introduction

Ectopic fat deposition refers to an excessive accumulation of lipids (mainly triglycerides) in nonadipose tissues, such as liver, muscle, and pancreas [1]. A large number of studies have shown that ectopic fat deposition is closely associated with insulin resistance (IR) and related metabolic diseases, including type 2 diabetes, atherosclerosis, and dyslipidemia [2]. However, its underlying mechanism has not yet been fully elucidated. In recent years, increasing evidence have shown that chronic low-grade inflammation is closely related to ectopic fat deposition and metabolic diseases; for example, elevated inflammatory factors are often observed in patients with ectopic fat deposition, such as fatty liver and fatty pancreas [3, 4]. Usually, this inflammatory condition is linked with overnutrition; however, a recent study reported elevated C-reactive protein (CRP) in nonobese or overweight subjects with nonalcoholic fatty liver disease, revealing that inflammation may play a critical and direct role, independent of excessive lipid from diet, in the development of ectopic fat accumulation [5]. Since ectopic fat accumulation involves both adipose tissue and nonadipose tissue, the purpose of the present review was to summarize the important evidence linking inflammation with ectopic lipid deposition in both adipose tissue and nonadipose tissue, in order to improve our understanding of the mechanism of ectopic lipid deposition.

## 2. Inflammation Causes Decreased Capacity of Fat Storage in Adipose Tissue

Adipose tissue is crucial for maintaining energy and metabolic homeostasis. One important function of adipocytes is to store TG, and the impairment of this function may have an effect on lipid handling in adipose tissue and, thereby, further contribute to excessive fat accumulation in nonadipose tissues. Many studies demonstrate that obese individuals often have enlarged adipocytes with overloaded lipid content and excess lipids “spill over” from the incompetent and dysfunctional adipose tissue, thereby exposing other tissues to an excessive influx of lipids, leading to ectopic fat deposition [6]. However, this “spill over” hypothesis cannot explain why some nonobese patients who are suffering metabolic disorders could also be accompanied by ectopic lipid accumulation. Besides, lipodystrophy, which is characterized by atrophic subcutaneous fat and IR, is often accompanied by ectopic fat accumulation in liver and/or in skeletal muscle. In mice models of lipodystrophy, transplantation of adipose tissue reversed IR and lipid content in both liver and skeletal muscle [7]. Furthermore, the development of lipodystrophy is correlated with the mutations of several genes participating in adipose metabolism, such as peroxisome proliferator-activated receptor *γ* (PPAR*γ*), 1-acylglycerol-3-phosphate-O-acyltransferase2 (AGPAT2), and Berardinelli-Seip congenital lipodystrophy (BSCL2), suggesting that this disease may be associated with adipocyte differentiation and lipid synthesis disorders in adipocytes [8]. Interestingly, lipodystrophic patients exhibited higher circulating concentrations of tumor necrosis factor-*α* (TNF-*α*) in adipose tissue, implying that inflammation may play an important role in the pathogenesis of the ectopic lipid deposition [9, 10].

Adipose tissue is composed of mature adipose cells and stroma-vascular fraction (SVF). In the SVF, mesenchymal stem cells as well as preadipocytes are able to differentiate into mature adipocytes for fat storage [11], and the whole process is controlled by some critical transcription factors and enzymes, including PPAR*γ*, CCAAT enhancer binding protein (C/EBP*α*), sterol regulatory element-binding protein 1 (SREBP-1), fatty acid synthase (FAS), acetyl-CoA carboxylase enzymes (ACC), and stearyl coenzyme A desaturase 1 (SCD-1) [12]. Gustafson and Smith [13] have shown that both interleukin-6 (IL-6) and TNF-*α* prevented the normal development of preadipocytes to fully differentiated adipose cells and lipid accumulation with decreased expression of PPAR*γ*2 and C/EBP*α*, which keep the cells undifferentiated. As an acute phase protein, serum amyloid A (SAA) is one of the most sensitive inflammatory markers, which is highly correlated with obesity, dyslipidemia, and insulin resistance. Remarkably depression of PPAR*γ*, C/EBP*β*, and C/EBP*α* was observed in preadipocytes treated with recombinant SAA (rSAA), leading to decreased intracellular lipid accumulation [14]. In Salles et al.'s study [15], after a 2-week high fat diet (HFD), TNF-*α*-knockout (TNF-*α*-KO) mice presented two-fold more adipose fat pad mass than control mice, while interestingly TNF-*α*-KO mice showed lower hepatic TG and ceramide accumulation in liver, with significantly declined adipose inflammatory markers, including resistin, monocyte chemotactic protein-1 (MCP-1), SAA3, and F4/80, implying decreased levels of inflammatory cytokines in adipose tissue might improve fat storage capacity of adipose tissue to prevent abnormal lipid deposition in nonadipose tissues.

Lipolysis of mature adipocytes is another important foundation for maintaining the balance of lipid metabolism in adipose tissue, which is conducted mainly by two rate-limiting enzymes, hormone-sensitive lipase (HSL) and fatty triglyceride lipase (ATGL) [16]. Except for impaired adipocyte differentiation, the inflammation-induced lipolysis may also be responsible for decreased lipid storage capacity of adipose tissue. In vitro, both IL-6 and TNF-*α* were proved to promote lipolysis in 3T3-L1 preadipocytes and increase free fatty acid in supernatant [13]. While in vivo, IL-6 injection also upregulated the level of fatty acid in serum. In morbidly obese patients, significantly elevated HSL and ATGL mRNA levels were observed, with enhanced serum CRP levels. Watt et al. [17] found that IL-6 administration promoted lipolysis with higher HSL mRNA and nonesterified fatty acid (NEFA) in serum. All evidence above supports that inflammation influences lipolysis by affecting the expression of HSL. However, some previous studies reported that TNF-*α* downregulated ATGL and HSL mRNA, without any changes in protein expression [18, 19]. Until recently, a study from Yang et al. [20] indicated that inflammation may stimulate basal lipolysis in adipocytes by regulating the function of ATGL. ATGL action in TNF-*α*-induced lipolysis was promoted by the depletion of G0/G1 switch protein 2 (G0S2) contents, which binds directly to ATGL and is capable of inhibiting its lipase activity. Yang found that TNF-*α* treatment inhibited the activity of ATGL by reducing both gene and protein expression of G0S2 to induce lipolysis in adipocytes, without any changes in expression of ATGL proteins. Remarkably, restoration of G0S2 protein levels by adenovirus-mediated ectopic expression was sufficient to prevent TNF-*α*-induced increase of glycerol release.

**Figure 1:**
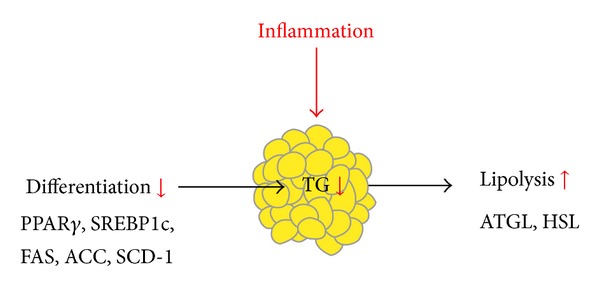
Inflammation decreases the lipid storage capacity of adipose tissue by inhibiting preadipocyte differentiation and increasing lipolysis.

Therefore, in terms of adipose tissue, inflammatory cytokines could decrease the lipid storage capacity by inhibiting preadipocytes differentiation and increasing lipolysis, which might further contribute to excessive fat accumulation in nonadipose tissues (Figure 1).

## 3. Effect of Inflammation on Nonadipose Tissue: Inflammation May Promote Fat Deposition in Nonadipose Tissue

### 3.1. Inflammation and Hepatic Fat Deposition

As a key metabolic organ, liver plays a crucial role in lipid metabolism. The balance of hepatic fat homeostasis depends on several pathways, influx of free fatty acids from adipose tissue due to lipolysis, de novo lipogenesis (DNL), fatty acid oxidation, and lipoprotein secretion [21]. Any abnormality of the processes above could contribute to lipid accumulation in liver.

Of the TG in liver, 59.0% derives from NEFAs, 26% from DNL, and 15% from the diet, which highlights the effect of fatty acid transportation on hepatic lipid content [22]. The transport of fatty acids into the liver is mediated via fatty acid transporters such as fatty acid transport protein (FATP), fatty acid translocase (FAT/CD36), fatty acid binding protein (FABP), and caveolin-1. Higher FAT/CD36 and FABP levels were observed in liver of nonalcoholic fatty liver disease (NAFLD) patients who are often accompanied with high levels of inflammatory factors, which were positively related to liver fat content [23, 24]. Furthermore, Margarita found that IL-6 induced higher FABP gene and protein expression in both HepG2 cells and primary mouse hepatocytes, leading to increased intracellular lipid content [25]. Moreover, in Salles et al.'s study [15], after a 12-week HFD, TNF-*α* KO mice showed significantly lower liver ceramide and TG content with an obviously decreased CD36 mRNA compared to their wide type (WT) counterparts. All the evidence above gives us a clue that chronic inflammation may increase hepatic fat accumulation by enhancing fatty acid uptake with upregulated FA transporters.

Lipogenesis in hepatocytes is under control of a series of critical genes, such as SREBP-1c, FAS, ACC, and SCD-1. Above all, SREBP-1c plays the key role for regulating the expression of genes encoding rate-limiting enzymes responsible for de novo lipogenesis, of which FAS and ACC seem to be particularly important. Several studies have discussed the effects of inflammation on SREBP1c. In recent years, our group has focused on the effect of inflammation on lipid metabolism in liver. We induced a chronic systemic inflammation by subcutaneous injection of 10% casein in C57BL/6J mice. Significant increases of IL-6, TNF-*α*, and SAA were observed in casein-injected mice compared with the respective controls, suggesting that chronic systemic inflammation was successfully induced in vivo. Our results showed that chronic systemic inflammation induced by casein injection exacerbated lipid accumulation in the liver of mice fed with normal chow diet (NCD) and HFD, with upregulated mRNA and protein expression of SREBP-1, ACC, and FAS in liver, which indicated that chronic systemic inflammation increased lipogenesis in liver, resulting in hepatic lipid deposition [26]. In vitro, we also found that inflammatory factor TNF-*α* raised the expression of SREBP-1c, FAS, and ACC in HepG2 hepatocytes, leading to enhanced lipid accumulation (data unpublished). Similar results were observed in L02 hepatocytes [27]. Studies above were together extracted to a hypothesis that inflammation may aggravate hepatic steatosis by promoting lipid synthesis. Accordingly, inhibiting inflammation could improve lipid content in liver. In HFD treated TNF-*α*−/− mice, lipid content in liver was obviously reduced, accompanied with declined protein expression of SREBP1c and FAS [15]. Moreover, in recent years, sirtuin1 (SIRT1) has been shown to be involved in the process of anti-inflammation. The activation of SIRT1 is proved to exert anti-inflammatory effects by inhibiting production of TNF-*α*, MCP-1, and IL-8 via blockade of nuclear factor-*κ*B (NF-*κ*B). Some studies reported that administration of SIRT1 activator ameliorated fat accumulation in the liver of monosodium glutamate (MSG) mice which exhibited obesity and IR, and the expression of lipogenic genes, such as SREBP-1c, FAS, and ACC, was reduced by SIRT1 activator treatment, with declined expression of inflammatory cytokines, which also provided evidence for the critical role of inflammation in hepatocytes lipogenesis [28, 29].

Most fatty acids are metabolized through *β*-oxidation, which occurs mainly in mitochondria. The oxidation of intrahepatocellular fatty acid is regulated by a variety of key enzymes, including PPAR*α*, PPAR*γ* coactivator 1*α* (PGC-1*α*), and carnitine palmitoyltransferase 1 (CPT1). In patients with NAFLD, the expression of PPAR*α* and CPT1 in liver was decreased, indicating impaired fatty acid oxidation [30]. Glosli et al. [31] found that in hTNF-*α* transgenic mice, hepatic triglycerides were enhanced and accompanied by reduced hepatic mRNA levels or activities of CPT-II, mitochondrial HMG-CoA synthase, peroxisome proliferator-activated receptor *α* (PPAR*α*), and fatty acyl-CoA oxidase (FAO). In addition, as previously described, SIRT1 activators could attenuate liver steatosis with ameliorated inflammation. While in liver-specific SIRT1 knockout mice, lipid content in liver was obviously elevated, with decreased PPAR*α* and PGC-1*α* and impaired fatty acid oxidation [32].

IL-6 is another critical inflammatory cytokine. However, the effect of IL-6 is still controversial. Long-term IL-6 injection ameliorates fatty liver of obese mice by stimulating hepatic fatty acid *β*-oxidation with increased hepatic PPAR*α* [33]. Intriguingly, treatment of cultured hepatocytes with various concentrations of IL-6 downregulates the expression of PPAR*α* [34, 35]. Matthews et al. [36] reported that mice with global deletion of IL-6 displayed hepatosteatosis, liver inflammation, and impaired whole-body insulin sensitivity when compared with control mice on a standard chow diet, revealing that IL-6 might be protective against hepatic steatosis and inflammation.

Hepatic TG could be delivered as VLDLs and secreted into circulation. The formation of VLDL involves the fusion of apoB with a TG droplet, which is mediated by microsomal triglyceride transfer protein (MTP). However, in patients with dyslipidemia, serum LDL-c and VLDL levels were observed to be positively associated with CRP and TNF-*α* [37]. Additionally, Pérez et al. reported that IL-6 treatment could upregulate hepatic apoB synthesis and secretion [38], implying a promoting effect of VLDL secretion of inflammation. This paradox might be explained since the lipid metabolism in liver is in a dynamic equilibrium and a fat deposition develops only when the inflammation induced fatty acid uptake or TG synthesis surpasses the inflammation-stimulated increase of hepatic lipid secretion.

**Figure 2:**
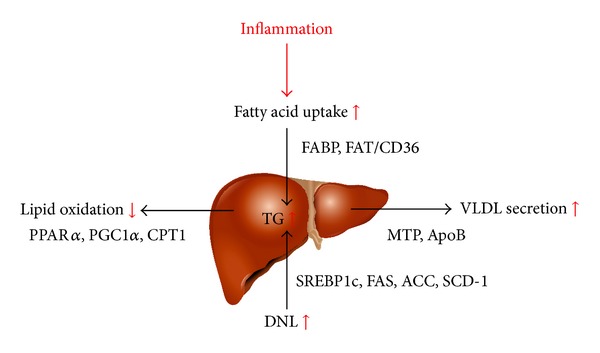
Inflammation promotes hepatic lipid accumulation through increased fatty acid uptake, enhanced TG synthesis, and reduced fatty acid oxidation.

It seems that the chronic low-grade inflammation, especially the proinflammatory cytokine TNF-*α*, might promote hepatic lipid accumulation through increased fatty acid uptake, enhanced TG synthesis, and reduced fatty acid oxidation in liver, and anti-inflammatory treatment may ameliorate this adverse effect. However, further studies are needed to investigate the effects of other different inflammatory cytokines on hepatic lipid accumulation (Figure 2).

### 3.2. Inflammation and Fat Deposition in Skeletal Muscle

Skeletal muscle is the major organ for fatty acid consumption, barely for lipid synthesis or storage. The increase of the lipid content in skeletal muscle which mainly results from increased fatty acid uptake and decreased *β*-oxidation can directly affect glucose and lipid metabolism and insulin sensitivity [39]. Several studies have indicated that inflammation may regulate fatty acid oxidation in skeletal muscles.

Increased lipid content as well as reduced *β*-oxidation of fatty acid and upregulated fatty acid uptake (a greater abundance of FAT/CD36) in muscle are observed in obese subjects who are characterized by inflammation state [40], indicating a negative role of inflammation on lipid accumulation in skeletal muscle. However, this view remains controversial, as in Salles et al.'s report [15] no significant difference of ceramide and TG concentrations was found in hind-limb skeletal muscles between TNF-*α*-KO and WT mice after a 2-week HFD. Furthermore, TNF-*α*-KO and WT mice showed similar levels of expression of genes involved in fatty acid oxidation, fatty acid uptake, or fatty acid synthesis, including PPAR*α*, CPT1*α*, CD36, SREBP1, and FAS. In vitro, TNF-*α* treatment was reported having no effect on FA oxidation but increased FA incorporation into diacylglycerol. Besides, IL-6 was identified to raise lipid oxidation via upregulation of PPAR*α*, PGC-1*α*, and PPAR*γ* mRNA expression by activating AMP-activated protein kinase (AMPK) signal pathway in skeletal muscle and decreasing fat accumulation in human primary skeletal muscle cells [41–45]. Those results propose a different role of inflammation on lipid metabolism in skeletal muscle from the role in liver.

### 3.3. Inflammation and Fat Deposition in Other Tissues

As we discussed above, inflammation is closely correlated with fat content in liver and skeletal muscle; however, should inflammation be responsible for lipid deposition in other tissues? Wan et al. reported that inflammatory cytokines TNF-*α* and IL-6 upregulated the expression of FAT/CD36 at both mRNA and protein levels and exacerbated intracellular lipid accumulation in human mesangial cells (HMCs) and renal tubular epithelial HK-2 cells, showing the effect of inflammation on lipid metabolism in kidney [46]. In vivo, casein injection significantly increased lipid deposition in kidneys in C57BL/6J mice, which suggested that inflammatory stress increased lipid accumulation in kidneys [47]. Cardiac adiposity, characterized by an increase in intramyocardial triglyceride content and an enlargement of the volume of fat surrounding the heart and vessels, was reported to be positively associated with inflammatory markers [48]. Pioglitazone, which has been proved to reduce inflammatory cytokines, was shown to reduce intramyocardial triglyceride content in T2D patients [49]. Besides, Ma et al. reported that after injection of 10% casein for 8 weeks, the high-fat-fed apolipoprotein 3 (Apo3) KO mice represented significantly increased plasma SAA levels as well as elevated lipid accumulation in cardiac blood vessels, which indicated that inflammatory stress may markedly exacerbate lipid accumulation in cardiac blood vessels [50]. In another study, thirty C57BL/6J lean controls and 30 leptin-deficient obese female mice were fed a 15% fat diet, and after 4 weeks, obese mice presented much higher TG content in pancreas compared with lean controls, with raised IL-1*β* and TNF-*α* concentrations. Those studies provide some clues that inflammation might promote lipid deposition in tissues such as kidney, heart, and pancreas; nevertheless, more evidence both in vivo and in vitro is still needed to further prove this effect [51].

## 4. Conclusion

**Figure 3:**
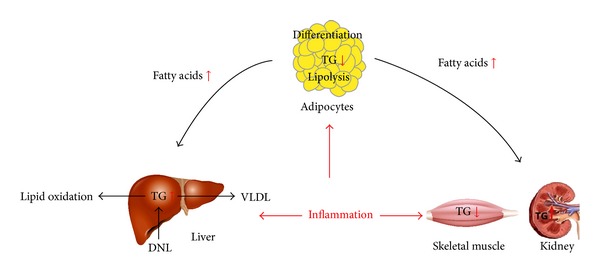
On one hand, chronic low-grade inflammation inhibits the differentiation of preadipocytes, increases lipolysis to upregulate the serum free fatty acid levels, and decreases the fat storage capacity of adipose tissue. On the other hand, inflammation directly influences lipid metabolism of liver, skeletal muscle, kidney, pancreas, and so forth, by different effects on process of ectopic fat deposition.

In summary, chronic low-grade inflammation plays an important role in the development of ectopic fat deposition. On one hand, inflammatory cytokines decrease the lipid storage by inhibiting the differentiation of preadipocytes and increasing lipolysis, leading to upregulated free fatty acid level in serum and abnormal fat accumulation in other tissues. On the other hand, inflammatory cytokines directly interrupt the lipid metabolism in nonadipose tissues. In liver, increased lipid accumulation results from elevated import of fatty acids, lipid synthesis, and declined fatty acid oxidation induced by inflammation. Some evidence, but not enough, reveals that inflammatory cytokines might promote lipid deposition in other tissues such as kidney, heart, and pancreas. Further researches are required to investigate the effects and mechanisms of inflammation on nonadipose tissues (Figure 3).
